# Reconstruction of molecular vibrational spectra from light–molecular vibration coupling spectra using deep learning

**DOI:** 10.1007/s44211-026-00933-x

**Published:** 2026-06-11

**Authors:** Yoshiaki Nishijima, Saoki Imai, Haruto Maeda

**Affiliations:** 1 Department of Electrical and Computer Engineering, Graduate School of Engineering, Yokohama National University, 79-5, Tokiwadai, Hodogaya-ku, Yokohama, Kanagawa 240-8501 Japan; 2 Institute of Advanced Sciences, Yokohama National University, 79-5, Tokiwadai, Hodogaya-ku, Yokohama, Kanagawa 240-8501 Japan; 3 Institute for Multidisciplinary Sciences, Yokohama National University, 79-5, Tokiwadai, Hodogaya-ku, Yokohama, Kanagawa 240-8501 Japan; 4 PRESTO, JST, Tokyo, Japan

**Keywords:** Mid infrared spectroscopy, metasurface, light-matter interaction

## Abstract

Light–molecular vibration coupling on metasurfaces induces complex optical phenomena, such as Fano resonance and Rabi splitting, which complicate molecular identification via conventional spectroscopic analysis. In this study, we developed an analytical system that uses deep feature learning to directly extract molecule-specific absorption information from these complex spectra. For a molecular vibration model with a single peak, we evaluated a DenseNet-169 convolutional neural network (CNN) model using 1,496 spectra generated via finite-difference time-domain (FDTD) simulations; however, the results were suboptimal. In contrast, for a two-peak molecular vibration model, we trained multiple CNN models, including DenseNet-169, on a dataset of 80,267 spectra. Consequently, we successfully reconstructed absorption coefficients with an extremely high accuracy, achieving a mean coefficient of determination ($R^2$) of 0.9209, even in complex systems with overlapping vibrational peaks. This approach demonstrates significant potential as a foundational technology for next-generation molecular sensing.

**Graphical abstract:**

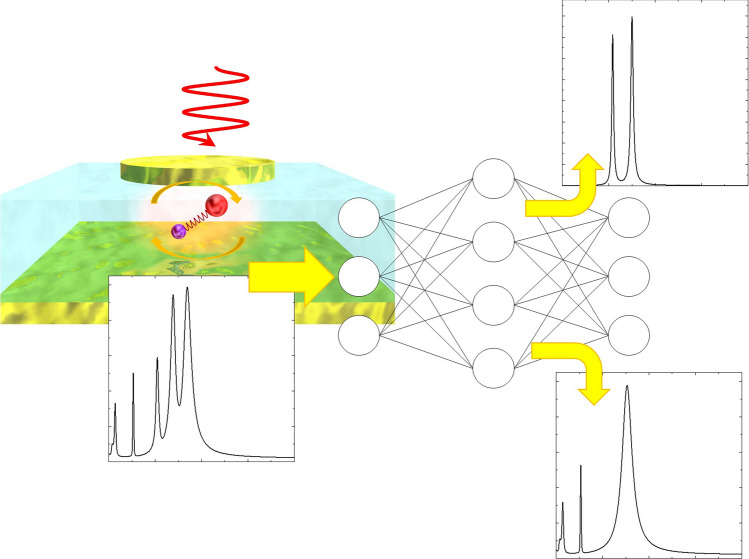

## Introduction

Mid-infrared (MIR) spectroscopy is widely utilized in chemistry, medicine, and environmental monitoring to capture unique vibrational modes, often referred to as molecular “fingerprints”. However, the absorption cross-section (molar extinction coefficient) in the MIR region is significantly smaller than that in the ultraviolet–visible (UV–Vis) region. Therefore, signal enhancement is essential for high-sensitivity detection. Surface-enhanced infrared absorption (SEIRA) is a well-established technique for enhancing infrared signals. In 2012, we demonstrated for the first time in the world that SEIRA arises from plasmonic resonances during extraordinary optical transmission in metal-hole arrays [1]. Since then, research into SEIRA using controlled plasmonic structures has intensified worldwide [2–5]. Optical gas sensing for the detection of trace volatile organic compounds (VOCs) has attracted particular attention as an application of this signal enhancement [6, 7]. To achieve higher sensitivity, stronger field enhancement is required. We have focused on metal-insulator-metal (MIM) nanostructures and found that strong light localization occurs within the dielectric layer [8–19]. Introducing molecules into this layer results in significant infrared absorption enhancement due to the intense, localized field. However, this also triggers phenomena such as Fano resonance and Rabi vacuum splitting resulting from strong light–molecular vibration coupling. Generally, interactions in an optical cavity are classified into weak- and strong-coupling regimes. If $\kappa$ is the photon decay rate of the cavity, $\gamma$ is the non-resonant decay rate, and *g* is the light–molecule coupling parameter, the interaction is considered strong when $g \gg \kappa , \gamma$, and weak when $g \ll \kappa , \gamma$. For example, SEIRA is a weak-coupling phenomenon [20].

These phenomena not only increase the intensity of infrared absorption but also exhibit complex behaviors, such as shifts in absorption wavelengths and merging with plasmon resonance spectra. This results in the loss of quantitative molecular information originally present in the infrared absorption spectra, which is a significant issue.

**Fig. 1:**
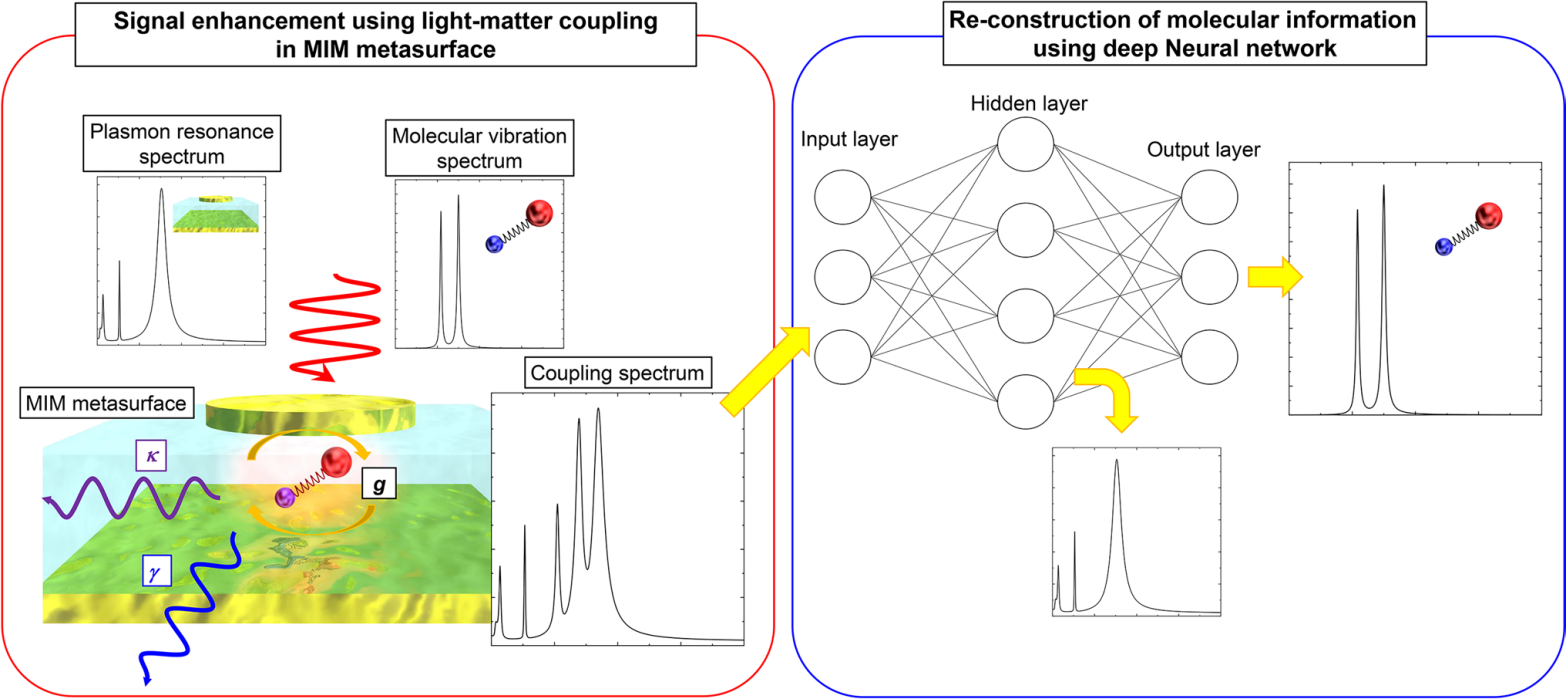
The conceptual figure of this research. We have established analytical methods of molecular vibrational modes from complex light-matter interactions

It is suggested that parameters representing the coupling state can be obtained by fitting with the Jaynes–Cummings model, as shown in Eq. 1 (Fig. 1) [21, 22].

1 $$\begin{aligned} R(\omega ) = \left| C \omega ^4 \frac{\omega _m^2 - \omega ^2 - i\gamma _m \omega }{(\omega _m^2 - \omega ^2 - i\gamma _m \omega )(\omega _c^2 - \omega ^2 - i\gamma _c \omega ) - 4\omega ^2 g^2} \right| ^2 \end{aligned}$$

Here, *C* represents the amplitude, $\omega$ is the angular frequency, $\omega _m$ is the center angular frequency of the molecular vibration, $\gamma _m$ is the line-width of the molecular vibration, $\omega _c$ is the center frequency of the plasmon resonance, $\gamma _c$ is the line-width of the plasmon resonance, and *g* is the coupling strength. This equation allows for effective fitting to determine the coupling coefficient, provided that the half-widths and center frequencies are somewhat predictable.

However, the large number of parameters makes it difficult to apply this model to systems with multiple peaks. Specifically, molecular vibrations consist of $3N-6$ for a non-linear shape molecule or $3N-5$ for a linear shape mode, where *N* is the number of atoms. Furthermore, plasmon resonances involve complex modes, such as diffraction gratings, higher-order modes, and dark plasmon modes. When molecular absorptions occur at adjacent frequencies, the coupled spectra interact, further complicating the overall spectral profile. Consequently, the fitting often fails to match the original spectrum perfectly, hindering the qualitative molecular evaluation.

Based on this background, this study aims to resolve complex physical spectra using the non-linear processing capabilities of deep learning. We constructed and evaluated a regression system to directly predict the "absorption coefficient (fingerprint)" for molecular identification by eliminating background metasurface characteristics from the input coupling spectra.

The concept of this study is illustrated in Fig. 1. We virtually simulated plasmon resonance and molecular absorption using FDTD calculations to comprehensively obtain coupling spectra under various parameters. These spectra were used as training data to investigate whether the original molecular absorption spectra could be reconstructed with high precision and to identify optimal conditions.

## Methods

### FDTD simulation for training data

The training data for deep learning were generated using the FDTD method via Ansys Lumerical FDTD solver. The structure consisted of a 200 nm Au film on a Si substrate, a 150 nm dielectric layer, and 50 nm thick Au nanodisks. The dielectric layer was assigned a complex permittivity $\varepsilon (\omega )$ based on a multi-peak Lorentz model, where $\varepsilon _\infty$ is the high-frequency permittivity, *i* is the imaginary unit, $\omega _n$ is the center angular frequency of the *n*-th peak, $\gamma _n$ is the half-width, and $f_n$ is the peak intensity, *f* represents the damping rate.

2 $$\begin{aligned} \varepsilon (\omega ) = \varepsilon _\infty + f\sum _{n=1}^{m} \frac{f_n \omega _n^2}{\omega _n^2 - \omega ^2 - i\gamma _n \omega } \end{aligned}$$

The simulation parameters were set as follows: $\varepsilon _\infty$: 2.25 (fixed) $\omega _n$: 1,800–2,500 $\text {cm}^{-1}$ (increment: 100 $\text {cm}^{-1}$) $\gamma _n$: 50–200 $\text {cm}^{-1}$ (increment: 50 $\text {cm}^{-1}$) *f*: 0.05 (fixed) $f_n$: 0.4–1.0 (increment: 0.2), *n*: 1 in single-peak model or 2 in two-peaks model. Nanodisk diameters ranged from 1,000 to 2,000 nm / 100 nm intervals, with the period fixed at twice the diameter in a hexagonal lattice. Normal incidence plane waves were used, and reflection spectra were monitored. Periodic boundary conditions were applied in the X and Y directions, with absorbing boundaries in the Z direction.

### Reconstruction of molecular vibrational spectra with deep learning

FDTD-derived data were converted into $224 \times 224$-pixel grayscale images with a 7-pixel line width for CNN application. For single-peak models, we fine-tuned a 169-layer DenseNet (DenseNet-169). Training, validation, and test data were split 8:1:1. For two-peak models, we utilized the ImageNet-1K dataset (ILSVRC 2012–2017) for pre-training, which includes approximately 1.28 million training images across 1,000 classes. We evaluated several models: VGG-19, ResNet, DenseNet-121, EfficientNet, ViT, and DenseNet-169 (7:1:2 split). Their characteristics are summarized in Table 1 [23–27]. We also compared DenseNet-169 with and without pre-training. Evaluation metrics included RMSE (Root Mean Square Error), MAE (Mean Absolute Error), and R2 Score, implemented in Python. RMSE measures the magnitude of error, with greater sensitivity to large outliers. MAE measures the average magnitude of errors in a set of predictions. R2 Score indicates how well the model explains the variability of the target data, where 1 represents a perfect fit All parameters are defined as below.

$$\begin{aligned} \text {RMSE} = \sqrt{\frac{1}{N} \sum _{i=1}^{N} (y_i - \hat{y}_i)^2} \end{aligned}$$

$$\begin{aligned} \text {MAE} = \frac{1}{N} \sum _{i=1}^{N} |y_i - \hat{y}_i| \end{aligned}$$

$$\begin{aligned} \text {R2 score} = 1 - \frac{\sum (y_i - \hat{y}_i)^2}{\sum (y_i - \bar{y})^2} \end{aligned}$$

where *N* is the number of data. we have 901 data in output layer. $\hat{y}$ is the prediction value, *y* is the correct value, $\bar{y}$ is the average of *y*. The average of these parameters has estimated as the evaluation value of the model.

**Table 1:** Deep learning models and characteristics

Model	Features
VGG-19	Simple stacking of $3 \times 3$ convolutions
ResNET	Utilizes skip connections to add input to output with 50–152 layers
EfficientNet	Optimizes depth, width, and resolution simultaneously with variable layers
ViT	Applies self-attention to image patches with variable layers
DenseNet-121	Dense connectivity where all 121 layers are linked to subsequent layers
DenseNet-169	More layers than DenseNet-121 with 169 layers

## Results

**Fig. 2:**
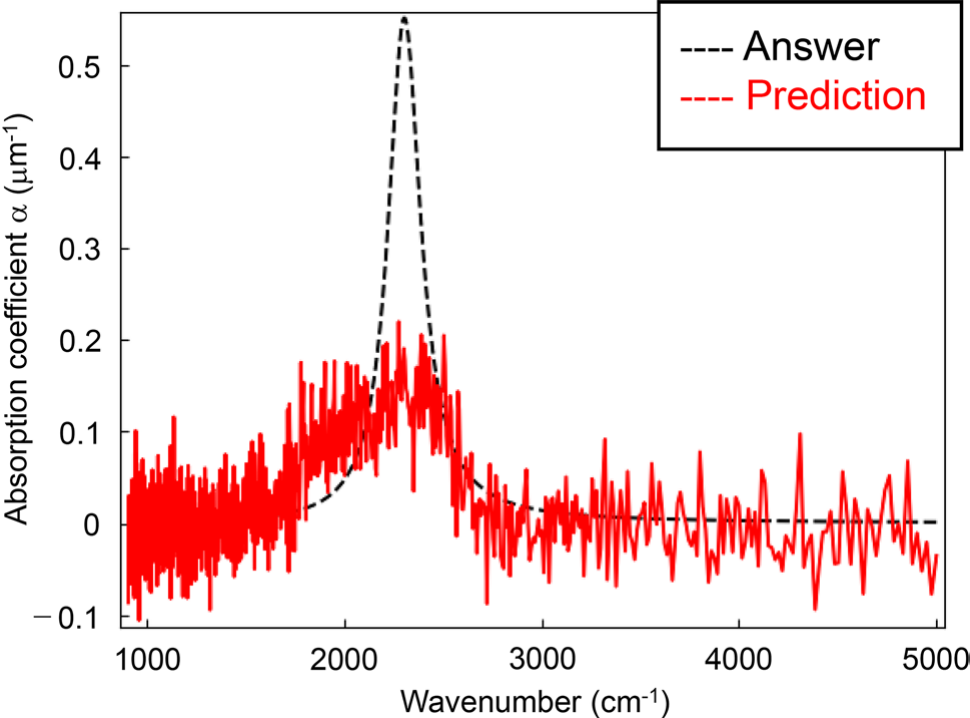
The result of reconstruction of molecular vibrational spectra in a single-peak model with 1419 training data

**Fig. 3:**
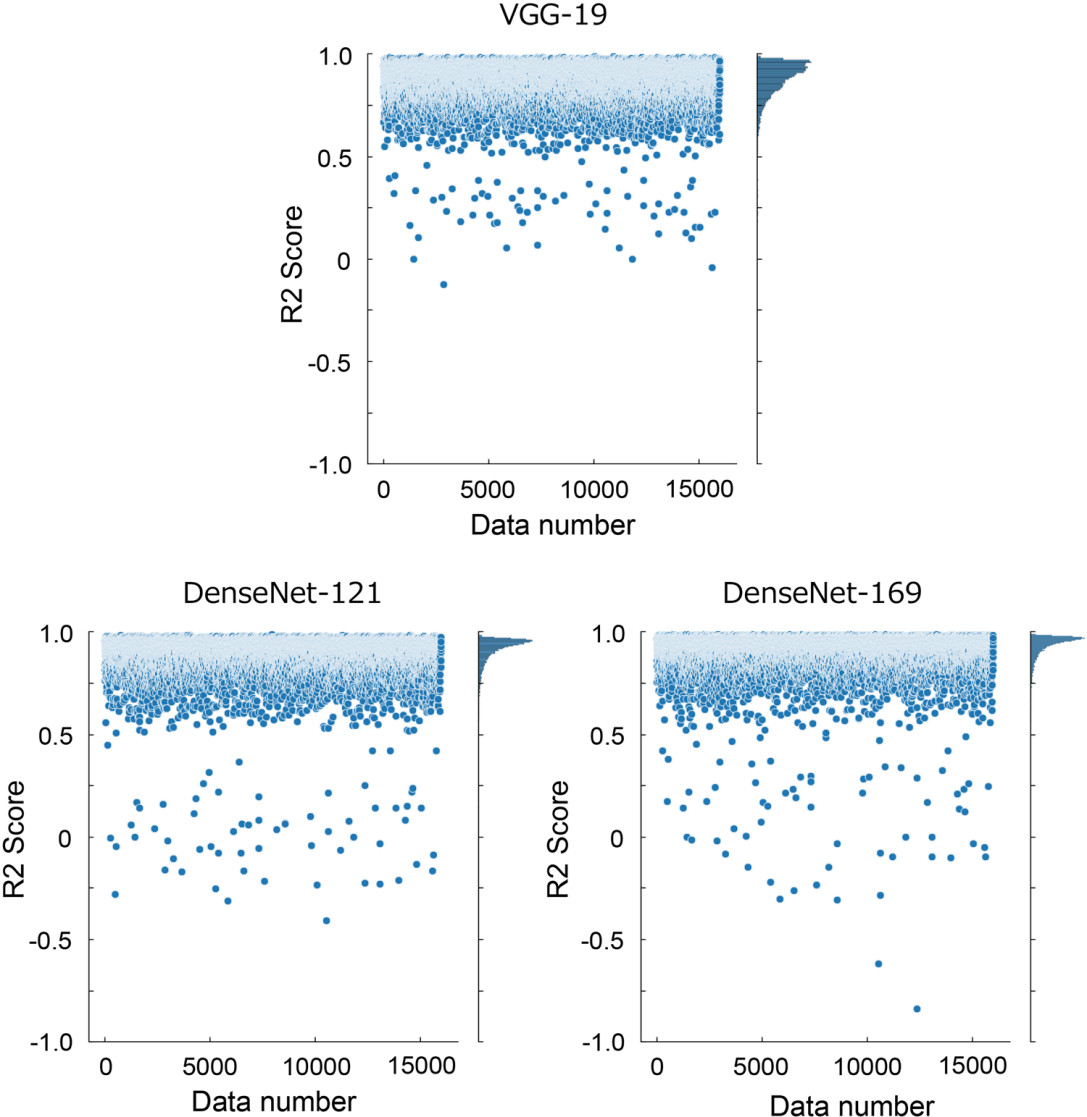
R2 Score in VGG-19, DenseNet-121, DenseNet-169

For the single-peak model using 1,419 training samples, the best R2 Score achieved was 0.3591. As shown in Fig. 2, while the peak position was roughly estimated, the absolute absorption coefficient was lower than the ground truth, the half-width was broader, and the signal-to-noise ratio (SNR) at the baseline was poor. This suggests that 1,419 samples were insufficient. Thus, we increased the dataset size by using a two-peaks model. Expanding the FDTD dataset to 80,267 samples, we evaluated multiple models. DenseNet-121 and DenseNet-169 yielded the best results, followed by VGG-19 (Table 2). Statistical analysis of R2 scores (Fig. 3) showed that DenseNet-169 had the most concentrated distribution, with a median of 0.94662 and a standard deviation of 0.08334.

The impact of pre-training was also significant. Without pre-training, the R2 score for DenseNet-169 dropped to 0.7642, confirming its necessity for high accuracy.

**Table 2:** Evaluation metrics for various models

Model	RMSE	MAE	R2 SCORE
VGG-19	0.04375	0.02028	0.8758
ResNET	0.04662	0.02761	0.8568
EfficientNet	0.08114	0.03468	0.6253
ViT	0.04253	0.02404	0.8760
DenseNet-121	0.03658	0.02158	0.9044
DenseNet-169	0.03256	0.01884	0.9209

**Fig. 4:**
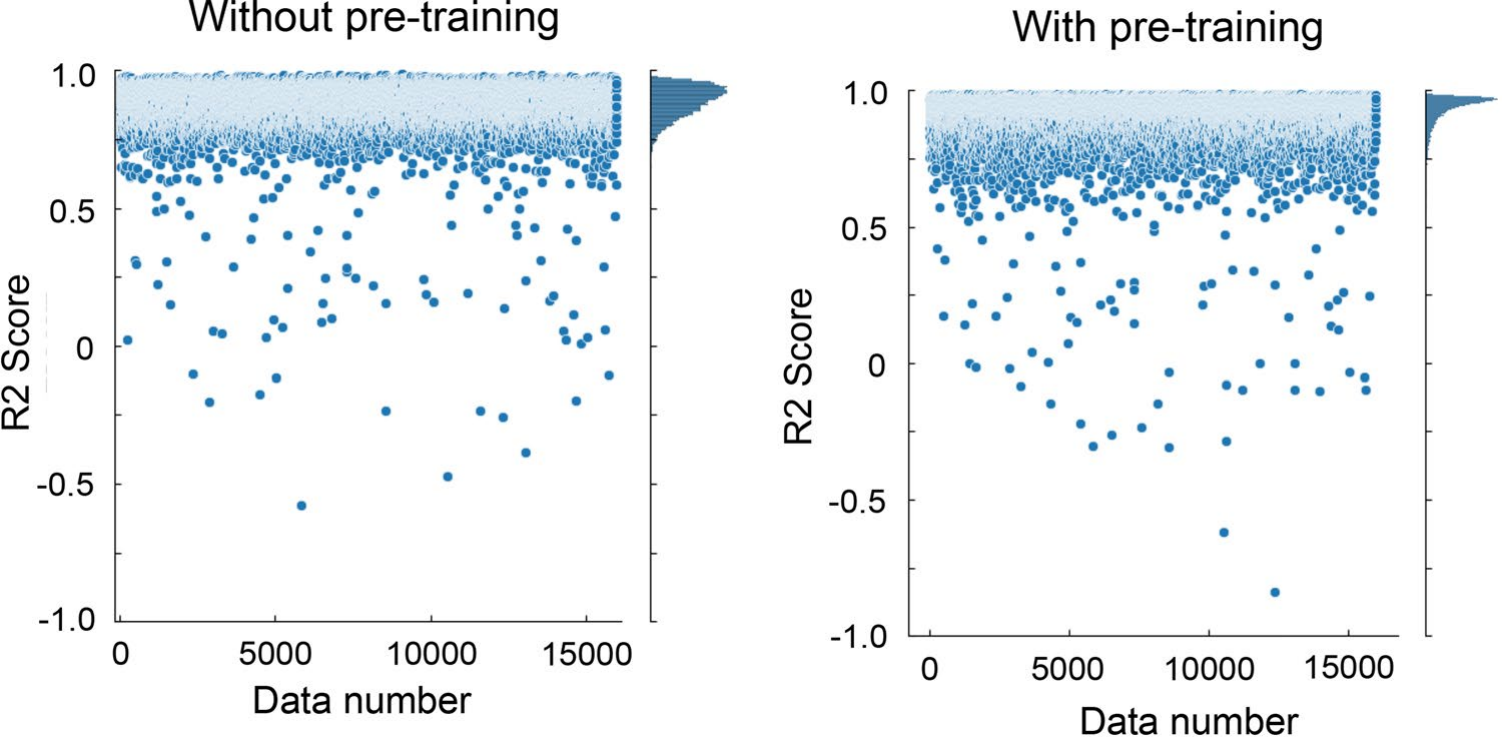
Deep learning result with/without pre-training. The figure of with pre-training was republished from figure 3

**Fig. 5:**
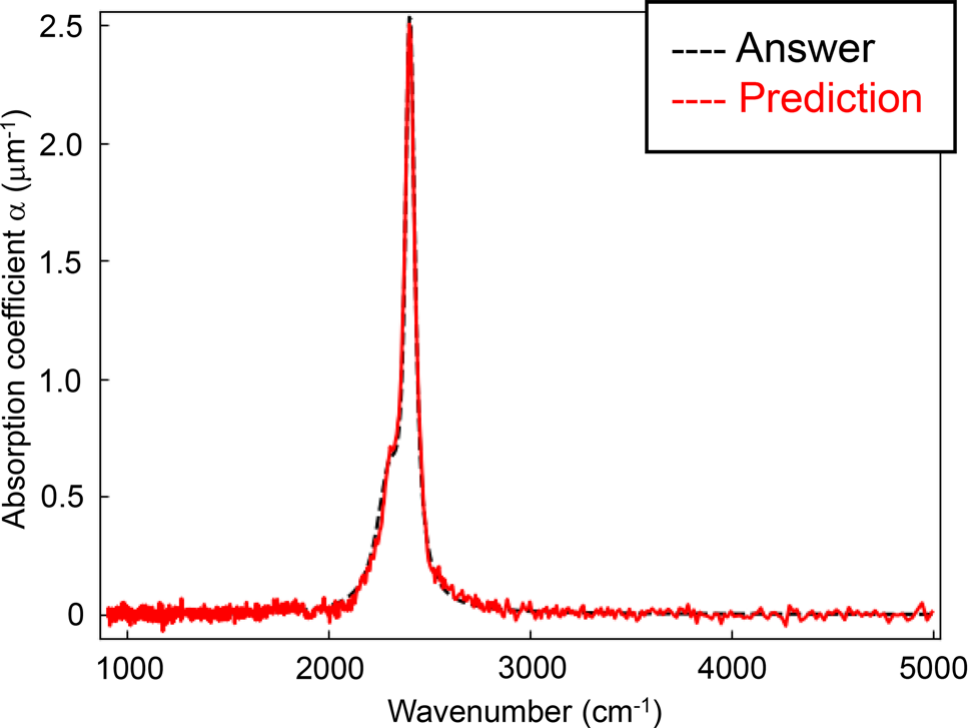
The best result spectra in re-constructing molecular vibrational spectra

The Impact of Pre-training with DenseNet-169 canbe estimated. In the case of Pre-training, as shown in Table 2, RMSE 0.03256, MAE 0.01884, and R2 Score 0.9209 have been obtained. However, in the condition of without Pre-training, RMSE 0.06193, MAE 0.03436, and R2 Score 0.7642 have been achieved. All the parameters deteriorated compared to pre-training Figs.4, 5.

In the most accurate case in DenseNet-169 model with pre-training, the best data in $R^2$ = 0.99909 has been obtained. The reconstructed spectra are shown in Fig. 5. The peak position error was within $5 \text { cm}^{-1}$. The model also accurately reconstructed the overlapping secondary peaks, demonstrating sufficient precision for chemical identification.

**Fig. 6:**
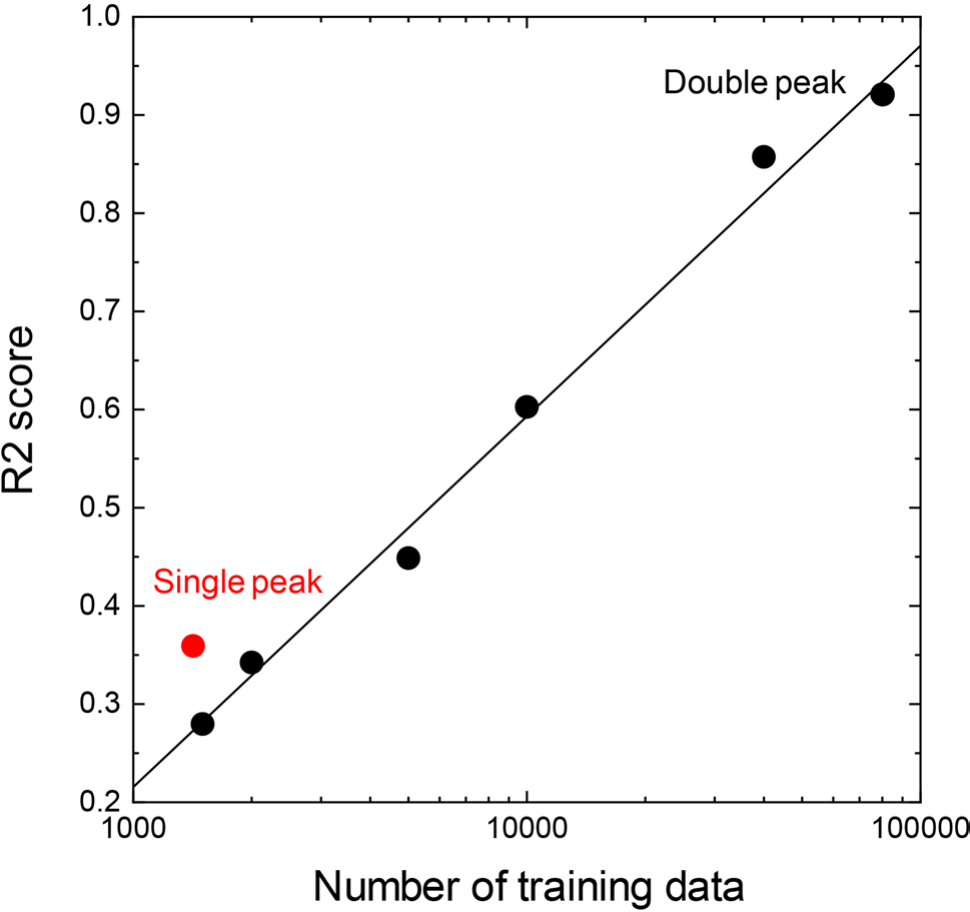
Relationship between the number of training samples and $R^2$ score. The solid line represents a linear regression fit obtained using the least squares method. Red plot comes from single peak data

To investigate whether the higher $R^2$ score of the double-peak model stems from the larger training dataset, we conducted a sensitivity analysis using randomly extracted subsets of 80,000, 40,000, 10,000, 5,000, 2,000, and 1,500 data points from the initial 80,267 samples. As shown in Fig. 6, the relationship between the number of training samples and performance exhibited excellent linearity on a logarithmic scale. The $R^2$ score at 1,500 samples (0.27966) was notably lower than that of the single-peak model (0.3591), thereby providing quantitative evidence that the expansion of training data is a key driver for the enhanced estimation precision.

## Conclusion

We proposed a new spectroscopic analysis method using deep learning. By applying image recognition technology, we automated and improved the precision of light–molecular vibration coupling spectral analysis, which previously required expert physical modeling. Future work will focus on validating robustness against experimental noise, analyzing systems with three or more peaks, and developing portable molecular sensing devices. Especially, in real molecular systems, the number of vibrational modes is determined by the number of constituent atoms *N*, specifically $3N-5$ for linear molecules and $3N-6$ for non-linear molecules. Consequently, light-molecule coupling spectra for actual compounds are expected to exhibit more complex profiles. While this study aimed to reconstruct molecular spectra using two peaks, further investigations with more peaks are required. An increase in the number of peaks, even when using the same parameters, would necessitate a larger volume of training data, which, in turn, is expected to enhance estimation accuracy. Furthermore, data augmentation techniques, such as using the first and second derivatives of the original spectral data, could improve estimation precision without increasing the number of raw training samples.

## Data Availability

Data underlying the results presented in this paper are not publicly available but may be obtained from the authors upon reasonable request.
